# Mindfulness-and body-psychotherapy-based group treatment of chronic tinnitus: a randomized controlled pilot study

**DOI:** 10.1186/1472-6882-12-235

**Published:** 2012-11-28

**Authors:** Peter M Kreuzer, Monika Goetz, Maria Holl, Martin Schecklmann, Michael Landgrebe, Susanne Staudinger, Berthold Langguth

**Affiliations:** 1 Department of Psychiatry and Psychotherapy, University of Regensburg, Universitaetsstrasse 84, Regensburg 93053, Germany

**Keywords:** Subjective tinnitus, Mindfulness-based therapy, Somatoform disorders, Self-management

## Abstract

**Background:**

Tinnitus, the perception of sound in absence of an external acoustic source, impairs the quality of life in 2% of the population. Since in most cases causal treatment is not possible, the majority of therapeutic attempts aim at developing and strengthening individual coping and habituation strategies. Therapeutic interventions that incorporate training in mindfulness meditation have become increasingly popular in the treatment of stress-related disorders. Here we conducted a randomized, controlled clinical study to investigate the efficacy of a specific mindfulness- and body-psychotherapy based program in patients suffering from chronic tinnitus.

**Methods:**

Thirty-six patients were enrolled in this pilot study. The treatment was specifically developed for tinnitus patients and is based on mindfulness and body psychotherapy. Treatment was performed as group therapy at two training weekends that were separated by an interval of 7 weeks (eleven hours/weekend) and in four further two-hour sessions (week 2, 9, 18 and 22). Patients were randomized to receive treatment either immediately or after waiting time, which served as a control condition. The primary study outcome was the change in tinnitus complaints as measured by the German Version of the Tinnitus Questionnaire (TQ).

**Results:**

ANOVA testing for the primary outcome showed a significant interaction effect time by group (F = 7.4; df = 1,33; p = 0.010). Post hoc t-tests indicated an amelioration of TQ scores from baseline to week 9 in both groups (intervention group: t = 6.2; df = 17; p < 0.001; control group: t = 2.5; df = 16; p = 0.023), but the intervention group improved more than the control group. Groups differed at week 7 and 9, but not at week 24 as far as the TQ score was concerned.

**Conclusions:**

Our results suggest that this mindfulness- and body-psychotherapy-based approach is feasible in the treatment of tinnitus and merits further evaluation in clinical studies with larger sample sizes.

The study is registered with clinicaltrials.gov (NCT01540357).

## Background

Tinnitus is defined as the perception of sound in the absence of an external sound source. About one in 10 adults is affected by chronic tinnitus, 20–30 percent of them dealing with considerable effects on daily living [1]. Severe tinnitus is frequently related to comorbidities such as insomnia [2], somatoform disorders, depression [3] or anxiety [4,5]. There have not been many effective treatment options up to now [6]. The best evidence is available for cognitive behavioral therapy so far, which aims to facilitate habituation by improving individual tinnitus coping strategies [6-8].

Mindfulness-based approaches use meditation techniques and yoga-like elements, and were introduced to clinical medicine about thirty years ago [9]. They have demonstrated efficacy in the treatment of a variety of stress-related disorders [10,11] such as anxiety and mood disorders [12] or chronic pain [13,14]. Very recently, mindfulness-based cognitive therapy has been proposed for the treatment of tinnitus [15-18]. Mindfulness also constitutes an integral element of the cognitive behavioral treatment programs for tinnitus that have shown convincing results in this indication so far [17,18].

In the current randomized waiting-list-controlled pilot study, we investigated a new manualized therapeutic approach, which is based on mindfulness- and body-psychotherapy and which has been specifically developed for the treatment of tinnitus patients (Tinnitus Atemtherapie) [19]. Essential components of the treatment program include mindfulness, meditation, self-massage, and breathing exercises. These components are intended to help patients use their inner resources to accept responsibility for themselves, become more self-sufficient and develop symptom acceptance.

## Methods

### Patient recruitment

Thirty-six patients with chronic tinnitus (duration ≥ 6 months) were randomized in an experimental group or a waiting list control group of 18 subjects, each after giving written, informed consent to the study. Randomization was conducted applying a computer-generated random list. Clients were recruited by direct referral from a local ENT physician and by an advertisement in the newsletter of the German Tinnitus League. The study was approved by the local ethics committee of the University of Regensburg, Germany (Ethikkommission der Fakultät für Medizin der Universität Regensburg). The study is registered with clinicaltrials.gov (NCT01540357).

### Study design

Inclusion criteria were 1) age between 18 and 80 years 2) location in the north-western part of Germany or in Belgium and the ability to understand the German language 3) no communicational problems 4) individual burden caused by subjective tinnitus for at least 6 months and 5) absence of any instable medical conditions. The experimental group was treated with the manualized group therapy; the control group was assessed at the identical time points during a waiting period of 24 weeks before they received treatment. No changes to methods and outcome measures were made after the trial began. Demographical and clinical characteristics of the enrolled patients are provided in Table 1.

**Table 1:** **Sample characteristics for the whole group and statistical parameters** (**mean** ± **standard deviation**)

	**Intervention group**	**Control group**	**Statistics**
n	18	18	
gender (male/ female)	11/7	8/10	χ^2^ = 1.0; df = 1; p = 0.317
age	49.6 ± 8.8	51.7 ± 16.0	t = 0.5; df = 34; p = 0.629
tinnitus duration	100.5 ± 119.1	142.3 ± 116.2	t = 1.1; df = 33; p = 0.300
number of previous treatments	3.4 ± 2.0	3.7 ± 2.1	t = 0.5; df = 33; p = 0.653
laterality (left, both/central, right)	6, 6, 5	8, 3, 7	χ^2^ = 1.6; df = 2; p = 0.451
**Questionnaires**			
Tinnitus Questionnaire	34.1 ± 15.8	37.4 ± 14.9	t = 0.7; df = 34; p = 0.512
Tinnitus Handicap Inventory	41.0 ± 20.4	45.9 ± 17.7	t = 0.8; df = 34; p = 0.448
Beck Depression Inventory	11.1 ± 8.1	11.8 ± 7.0	t = 0.3; df = 34; p = 0.793
**Numeric rating scales**			
loudness	5.7 ± 2.5	6.5 ± 2.2	t = 1.0; df = 34; p = 0.328
annoyance	7.2 ± 2.3	5.9 ± 3.0	t = 1.4; df = 33; p = 0.158
discomfort	6.9 ± 2.8	7.3 ± 2.3	t = 0.5; df = 34; p = 0.600
distractibility	6.2 ± 2.7	6.4 ± 2.7	t = 0.3; df = 34; p = 0.806
unpleasantness	5.9 ± 2.8	7.1 ± 2.2	t = 1.4; df = 34; p = 0.169
**Other** (**no**, **yes**)			
temporomandibular joint disorder	12, 5	14, 4	χ^2^ = 0.2; df = 1; p = 0.627
neck pain	7, 10	6, 12	χ^2^ = 0.2; df = 1; p = 0.631
other pain	10, 6	10, 8	χ^2^ = 0.2; df = 1; p = 0.681
influence of neck movement	12, 4	9, 9	χ^2^ = 2.2; df = 1; p = 0.134
psychiatric comorbidity	12, 5	14, 4	χ^2^ = 0.2; df = 1; p = 0.627

### Treatment program

The treatment program includes mindfulness, meditation, self-massage, and breathing exercises as the main components and was developed and manualized [19] by one of the authors (MH). MH is an experienced therapist with a focus on stress-related disorders and also conducted the therapeutic meetings in Aachen, Germany.

Briefly, the treatment consists of (1) meditation elements, (2) imagination exercises, (3) self-massage and individualized gentle movement exercises of the body, (4) exercises aiming at directing moment-to-moment awareness of body- and self- perception and (5) breathing exercises with emphasis on expiration in order to reduce muscle tension and increase relaxation. Participants were taught the therapeutic modules at two weekends (11 hours of treatment/weekend) with an interval of 7 weeks. Two weeks after each weekend and 11 and 15 weeks after the second training weekend patients gathered for a review meeting lasting 2 hours each. Patients were strongly encouraged to perform exercises themselves regularly and were instructed to contact and motivate each other by telephone at least once a week. Data assessment and analysis took place at the University of Regensburg, Regensburg, Germany.

### Assessment instruments and statistical analysis

**Figure 1:**
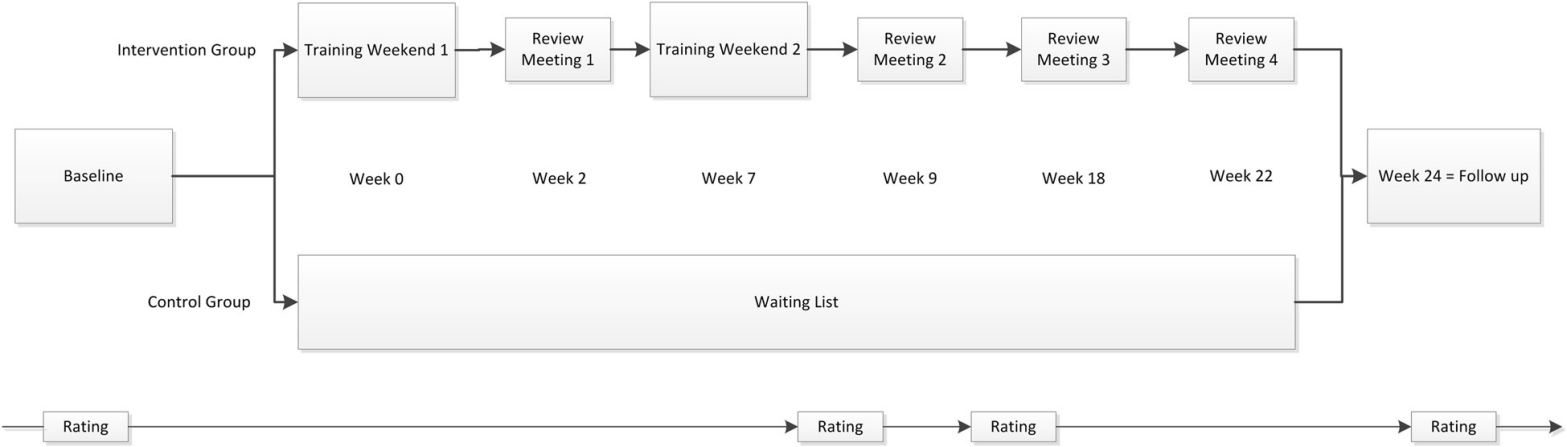
Structure and schedule of the study.

Tinnitus characteristics were assessed before treatment (baseline), at week 7 (end of second training weekend), at week 9 and week 24 (Figure 1). Tinnitus assessments included the German versions of the Tinnitus Handicap Inventory [20], the Tinnitus Questionnaire [21], the Beck Depression Inventory [22], several tinnitus numeric rating scales (loudness, discomfort, annoyance, distractibility, unpleasantness) [23]. Prospective assessment of treatment effects was performed using standardized procedures as established in the Tinnitus Research Initiative (TRI) database [23]. Data management was conducted according to the Data Handling Plan (TRI-DHP Version 06, May 9^th^, 2011). Data analysis was conducted according to the Standard Operating Procedure (TRI-SA Version 01, May 9^th^, 2011), which followed a study-specific Statistical Analysis Plan (SAP-002). All documents are to be found under http://database.tinnitusresearch.org/.

The primary outcome was the change in TQ scores from baseline to week 9. For this purpose, we conducted an analysis of variance (ANOVA) with the within-subjects factor time (screening and week 9) and the between-subjects factor group (intervention vs. control group). Repeated measures ANOVA as used in this study may be confounded with several issues, i.e., low statistical power, regression to the mean due to baseline differences, inflation of type I error due to multiple post hoc tests, etc. [24-26]. Thus, we controlled for these issues by repeating the primary outcome analysis by calculating an ANCOVA with the between-subjects factor group (intervention vs. control group), with the baseline values of the TQ as covariate, and with TQ score at week 9 as dependent variable. Regression slopes between the covariate and the dependent variable were comparable across conditions (0.848 and 0.864). Secondary outcome measures included chi-square tests for the variables group and treatment response, which was defined as amelioration of at least 5 points in the TQ [27]. In addition, we conducted an additional ANOVA with the factor group and time, this time including four measurement time points (screening, week7, week 9, and week 24). For THI, the numeric rating scales and BDI we performed identical ANOVAs.

## Results

The trial was conducted and terminated according to the study protocol priorily defined. No adaption was necessary during the course of the study (April 2010 to December 2010). The groups did not differ significantly in demographical or clinical characteristics as shown in Table 1.

Three participants did not complete the study procedures. One patient refused to participate after being randomized into the waiting list group; two patients did not return the follow-up-questionnaires after having participated in the meetings without giving any further explanation. These three patients were excluded from our analysis.

**Figure 2:**
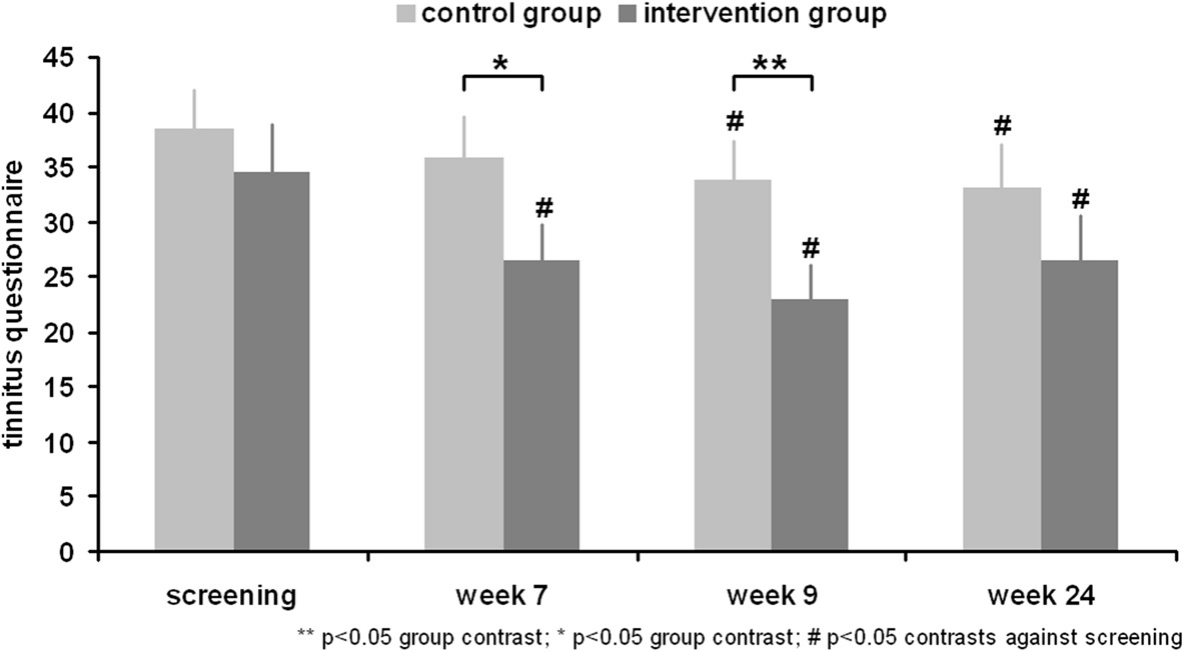
Tinnitus questionnaire.

ANOVA testing for primary outcome demonstrated a significant interaction effect time by group (F = 7.4; df = 1,33; p = 0.010; η^2^ = 0.183). Post hoc t-tests indicated an amelioration of TQ scores from baseline to week 9 in both groups (intervention group: t = 6.2; df = 17; p < 0.001; d = 1.458; control group: t = 2.5; df = 16; p = 0.023; d = 0.611) (Figure 2), but the intervention group improved at a higher rate than the control group (baseline: t = 0.7; df = 31; p = 0.480; d = 0.249; week 9: t = 2.2; df = 31; p = 0.036; d = 0.764). To account for potential statistical bias (see methods) we redid the primary outcome analysis with an ANCOVA using the baseline values as covariate and TQ score as single dependent variable. Group main effect was again significant (F = 10.948; df = 1,32; p = 0.002; η^2^ = 0.255). The responder rate was higher in the intervention group (14 responders out of 16 subjects) compared to the control group (8 responders out of 17 subjects; χ^2^ = 6.1; df = 1; p = 0.014) with an odds ratio of 7.9 (CI: 1.4-45.8) and a relative risk of 3.5 (CI: 1.0-12.8).

ANOVA with four measurement time points showed a nearly significant interaction effect time by group (F = 2.2; df = 3,93; p = 0.094; η^2^ = 0.066). Post hoc tests indicated a significant amelioration at week 7, week 9, and week 24 in contrast to baseline for the intervention group (all ts > 3.1; df = 15; all ps < 0.008; all ds > 0.766) and at week 9 and 24 for the control group in contrast to baseline (all ts > 2.5; df = 16; all ps < 0.023; all ds > 0.611). The groups differed at week 7 (t = 1.8; df = 31; p = 0.077; d = 0.638) and 9 (see primary outcome), but not at week 24 (t = 1.2; df = 31; p = 0.257; d = 0.402).

Concordant effects were also found for THI and BDI (see Table 2). Numeric rating scales did not show significant interaction effects (see Table 2). Means and standard deviations are provided for all measurements and assessment points in Table 2.

**Table 2:** **Sample characteristics over all measurement time points and statistical parameters** (**mean** ± **standard deviation**); **n**.**d**. = **not done**

		**Intervention group (df = 15)**	**Control group (df = 16)**	**Intervention vs**. **control group (df = 31)**	**Time by group interaction effect (df = 3,93)**
**Questionnaires**					
Tinnitus Questionnaire	screening	34.6 ± 16.7	38.5 ± 14.7	t = 0.7; p = 0.480	F = 2.2; p = 0.094
	week 7	26.4 ± 13.6	35.8 ± 15.5	t = 1.8; p = 0.077	
	week 9	22.9 ± 13.0	33.7 ± 15.2	t = 2.2; p = 0.036	
	week 24	26.5 ± 16.3	33.1 ± 16.6	t = 1.2; p = 0.257	
	week 7 vs. baseline	t = −4.3; p < 0.001	t = −1.4; p = 0.182		
	week 9 vs. baseline	t = −5.4; p < 0.001	t = −2.5; p = 0.023		
	week 24 vs. baseline	t = −3.1; p = 0.008	t = −2.7; p = 0.015		
Tinnitus Handicap Inventory	screening	40.9 ± 21.7	47.1 ± 17.5	T = 0.9; p = 0.373	F = 4.4; p = 0.006
	week 7	29.3 ± 17.1	44.8 ± 19.9	T = 2.4; p = 0.022	
	week 9	26.3 ± 17.4	41.4 ± 20.0	T = 2.3; p = 0.027	
	week 24	27.3 ± 19.9	41.3 ± 21.1	T = 2.0; p = 0.058	
	week 7 vs. baseline	t = −4.2; p < 0.001	t = −1.3; p = 0.225		
	week 9 vs. baseline	t = −7.4; p < 0.001	t = −3.3; p = 0.004		
	week 24 vs. baseline	t = −5.5; p < 0.001	t = −2.3; p = 0.037		
Beck Depression Inventory	screening	11.4 ± 8.4	12.3 ± 6.9	t = 0.4; p = 0.732	F = 4.4; p = 0.006
	week 7	8.7 ± 7.6	12.5 ± 6.6	t = 1.5; p = 0.135	
	week 9	6.3 ± 4.8	12.1 ± 6.7	t = 2.9; p = 0.007	
	week 24	7.6 ± 5.7	13.3 ± 8.7	t = 2.2; p = 0.035	
	week 7 vs. baseline	t = −2.6; p = 0.019	t = 0.4; p = 0.718		
	week 9 vs. baseline	t = −3.8; p = 0.002	t = −0.2; p = 0.841		
	week 24 vs. baseline	t = −2.5; p = 0.024	t = 0.8; p = 0.455		
**Numeric rating scales**					
loudness	screening	5.9 ± 2.5	6.7 ± 2.1	n.d.	F = 1.9; p = 0.135
	week 7	5.3 ± 2.8	6.6 ± 2.4	n.d.	
	week 9	5.1 ± 2.6	6.8 ± 2.1	n.d.	
	week 24	5.1 ± 2.7	7.0 ± 2.3	n.d.	
	week 7 vs. baseline	n.d.	n.d.		
	week 9 vs. baseline	n.d.	n.d.		
	week 24 vs. baseline	n.d.	n.d.		
annoyance	screening	6.2 ± 3.1	7.4 ± 2.3	t = 1.3; p = 0.225	F = 2.3; p = 0.087
	week 7	5.0 ± 2.7	6.9 ± 2.4	t = 2.2; p = 0.037	
	week 9	4.6 ± 2.2	7.4 ± 2.1	t = 3.6; p = 0.001	
	week 24	5.4 ± 2.6	7.2 ± 2.5	t = 2.1; p = 0.045	
	week 7 vs. baseline	t = −2.3; p = 0.037	t = −1.6; p = 0.110		
	week 9 vs. baseline	t = −2.5; p = 0.025	t = 0		
	week 24 vs. baseline	t = −1.5; p = 0.154	t = −0.4; p = 0.683		
discomfort	screening	7.1 ± 2.8	7.6 ± 2.0	n.d.	F = 1.9; p = 0.142
	week 7	5.3 ± 2.8	7.1 ± 2.3	n.d.	
	week 9	5.5 ± 2.7	7.4 ± 1.8	n.d.	
	week 24	5.6 ± 2.9	7.3 ± 1.9	n.d.	
	week 7 vs. baseline	n.d.	n.d.		
	week 9 vs. baseline	n.d.	n.d.		
	week 24 vs. baseline	n.d.	n.d.		
distractibility	screening	6.4 ± 2.8	6.6 ± 2.6	n.d.	F = 1.9; p = 0.132
	week 7	5.7 ± 3.0	6.8 ± 3.1	n.d.	
	week 9	5.3 ± 2.8	7.2 ± 2.1	n.d.	
	week 24	5.6 ± 2.7	7.2 ± 2.8	n.d.	
	week 7 vs. baseline	n.d.	n.d.		
	week 9 vs. baseline	n.d.	n.d.		
	week 24 vs. baseline	n.d.	n.d.		
unpleasantness	screening	6.2 ± 2.8	7.0 ± 2.2	n.d.	F = 1.1; p = 0.370
	week 7	5.4 ± 2.7	6.9 ± 2.5	n.d.	
	week 9	5.4 ± 2.5	7.0 ± 2.2	n.d.	
	week 24	5.4 ± 2.4	7.4 ± 2.0	n.d.	
	week 7 vs. baseline	n.d.	n.d.		
	week 9 vs. baseline	n.d.	n.d.		
	week 24 vs. baseline	n.d.	n.d.		

The responder rate at week 24 was not statistically higher in the intervention group (11 responders out of 16 subjects) compared to the control group (10 responders out of 17 subjects; χ^2^ = 0.4; df = 1; p = 0.554) with an odds ratio of 1.5 (CI: 0.4-6.5) and a relative risk of 1.3 (CI: 0.6-2.8).

## Discussion

The main finding of this pilot study was a significant reduction in the TQ score (baseline vs. week 9) after thirty hours of this new manualized group therapy compared to the waiting list control group. Very recently, first results were presented from a randomized controlled study investigating mindfulness-based therapy in tinnitus patients [16]. Notably, in this previous study neither mindfulness-based therapy nor relaxation training as a control condition exerted an immediate effect on tinnitus symptoms. This might at least in part be due to the large effect already yielded by the preceding psycho-education [16]. However, during the follow-up period mindfulness-based treatment was superior to relaxation therapy [16].

In contrast to this previous study [16], progress tended to stagnate in our study. Clearly, mindfulness-based therapy depends on practice behavior [16]. Thus, the retrogressive effects in our study point to a reduced training maintenance at follow-up and may indicate the relevance of boosting sessions for stabilizing treatment effects.

A pronounced long-lasting effect was also observed in a randomized, controlled study treating chronic tinnitus with a training program lasting five weeks with Qigong [28]. Notably, Qigong had been trained in sessions of 2 hours weekly and one could question the time schedule of two weekends of intensive training of up to 11 hours in our study. Possibly a longer-lasting and repetitive training process would enhance longer-lasting effects through better incorporation of the exercises into daily routine.

Mindfulness-based therapeutic approaches have demonstrated beneficial effects in other stress-related disorders such as chronic pain [13,29] or anxiety [12]. With effect sizes of 0.37 for pain scores [29] and between 0.24 to 0.47 on anxiety scores in patients with chronic medical diseases [10], the effects of mindfulness-based therapies were smaller in those studies when compared to the effect size of 0.80 in our study. However caution is warranted in such comparisons, since further data from larger samples will be needed before the effect size of mindfulness- and body-psychotherapy based treatment in tinnitus can be reliably estimated.

Future studies should also address the specific relevance of the different therapeutic components and their potential neurobiological mechanisms. A significant increase in alpha power through meditation has been revealed by electroencephalographic [30] and magnetencephalographic [31] studies. Alpha power in sensory areas is considered an indicator of inhibitory function [32] and an increase of alpha activity by neurobiofeedback or transcranial magnetic stimulation has been shown to result in reduced tinnitus perception [33,34]. Thus it is tempting to speculate that the beneficial effects of mindfulness- and meditation- based therapy may be mediated by an increase in alpha power. Moreover, brain areas which are known to be involved in tinnitus such as the left hippocampus [35], the posterior cingulate cortex [36], the temporo-parietal junction [37], and the cerebellum [38] have recently been shown to be altered by mindfulness meditation [39].

From a psychological point of view, few studies have focused on the potential mechanisms of mindfulness and acceptance-based training procedures in tinnitus patients, unfortunately. It has been proposed that mindfulness based therapy can facilitate adaptive responses by enhancing cognitive defusion strategies and symptom acceptance connected with a non-evaluative observation of internal events. For example, in a sample of 19 tinnitus patients the frequency of cognitive defusion behaviors and peak level of cognitive defusion as well as peak level of acceptance rated in session 2, predicted a symptom reduction 6 months following treatment, indicating that clients’ in-session acceptance and cognitive defusion behaviors appear to play an important role in the reduction of the negative impact of tinnitus [7].

## Conclusions

We are well aware of the limiting factors of the pilot study conducted, such as the small sample size, the inherent problems of waiting-list control conditions [40], and the fact that treatment effects may depend on the instructor, which might limit generalization of the results. Nevertheless our pilot data indicate the promise of mindfulness- and body-psychotherapy-based therapy in the treatment of tinnitus and warrant further investigation of its clinical and neurobiological effects in larger studies.

## Competing interests

MH has written the treatment manual for “Tinnitus Atemtherapie” and offers this treatment in private practice. MH has been supported by a grant from the Bundesverband der Innungskrankenkassen (IKK), Association of Health Insurances. The other authors declare no competing interests in relation to this article.

## Authors’ contributions

PK, SS; ML, MS and BL designed the study. MG and MH were responsible for the generation of the patients’ enrollment, randomized assignment of participants to the interventions and the practical conduction of participants’ training. SS and MG were responsible for data management and data entry in the TRI database. MS was responsible for statistical analysis. PK, MG, MS and BL drafted the manuscript. All authors approved the final version of the manuscript. For full trial protocol please contact the corresponding author.

## Financial disclosure

MH has written a book describing the methods of the applied behavioral techniques. The study has been financially supported by a grant from the Bundesverband der Innungskrankenkassen (IKK), Association of Health Insurances. The other authors have no conflicts of interest or disclosures to declare in relation to this article.

## Pre-publication history

The pre-publication history for this paper can be accessed here:

http://www.biomedcentral.com/1472-6882/12/235/prepub
